# p53 Activation following Rift Valley Fever Virus Infection Contributes to Cell Death and Viral Production

**DOI:** 10.1371/journal.pone.0036327

**Published:** 2012-05-04

**Authors:** Dana Austin, Alan Baer, Lindsay Lundberg, Nazly Shafagati, Annalise Schoonmaker, Aarthi Narayanan, Taissia Popova, Jean Jacques Panthier, Fatah Kashanchi, Charles Bailey, Kylene Kehn-Hall

**Affiliations:** 1 National Center for Biodefense and Infectious Diseases, School of Systems Biology, George Mason University, Manassas, Virginia, United States of America; 2 Mouse Functional Genetics, Institut Pastuer, Paris, France; The University of Texas Medical Branch, United States of America

## Abstract

Rift Valley fever virus (RVFV) is an emerging viral zoonosis that is responsible for devastating outbreaks among livestock and is capable of causing potentially fatal disease in humans. Studies have shown that upon infection, certain viruses have the capability of utilizing particular cellular signaling pathways to propagate viral infection. Activation of p53 is important for the DNA damage signaling cascade, initiation of apoptosis, cell cycle arrest and transcriptional regulation of multiple genes. The current study focuses on the role of p53 signaling in RVFV infection and viral replication. These results show an up-regulation of p53 phosphorylation at several serine sites after RVFV MP-12 infection that is highly dependent on the viral protein NSs. qRT-PCR data showed a transcriptional up-regulation of several p53 targeted genes involved in cell cycle and apoptosis regulation following RVFV infection. Cell viability assays demonstrate that loss of p53 results in less RVFV induced cell death. Furthermore, decreased viral titers in p53 null cells indicate that RVFV utilizes p53 to enhance viral production. Collectively, these experiments indicate that the p53 signaling pathway is utilized during RVFV infection to induce cell death and increase viral production.

## Introduction

Rift Valley fever virus (RVFV) is a *Phlebovirus* in the family *Bunyaviridae* that is transmitted primarily by mosquitoes and is emerging as a serious viral zoonosis affecting both livestock and humans. RVFV remains endemic in sub-Saharan Africa and has caused major outbreaks throughout Africa in the last century and recently in parts of the Arabic peninsula [1], [2], [3]. In animals, it is usually transmitted through the bite of a mosquito. Transmission to humans mainly occurs through close contact with infected animal tissue or body fluids as well as through aerosolization [3], [4]. In humans, it typically causes an acute mild febrile illness resembling the flu. However, in a small percentage of patients it results in serious clinical manifestations such as retinal lesions, meningoencephalitis, hepatitis, severe hemorrhagic fever, and death [1], [2], [3]. In livestock, RVFV causes vast economic devastation through both mortality of adult animals as well as an extremely high rate of abortion and fetal deformities [5]. Of particular concern are recent epidemiological surveys of RVFV outbreaks in humans that have shown a higher percentage of mortality among infected individuals, increasing from 2% to 45%, suggesting the virus could be evolving mechanisms of increased virulence and greater pathogenicity [6].

Not unlike other bunyaviruses in its family, RVFV is a single stranded RNA virus with a tri-segmented genome; the large (L) segment, Medium (M) segment and Small (S) segment [1]. The viral RNA dependent RNA polymerase (L protein) is encoded on the L segment [7], while the M segment codes for the precursor for two glycoproteins, Gc and Gn, as well as the nonstructural proteins, NSm (NSm2), 78 kDa (NSm1), and 73–75 kDa (NSm2-Gn) [8], [9], [10], [11]. The S segment codes for one structural protein, the nucleocapsid protein, N, and a nonstructural protein called NSs [12]. The nonstructural proteins are not essential for replication of the virus; however they do play a significant role in the pathogenesis of the disease *in vivo* [4]. NSs protein has been established as a virulence factor due to its ability to suppress the host’s immune response by counteracting the antiviral interferon (IFN) response [13], [14], [15], [16]. NSm has also been described as a virulence factor through anti-apoptotic functions by blocking caspase-3 and its downstream effectors, as well as initiator caspases, caspase-8 and 9 [17].

Our previous studies using reverse-phase protein microarray analysis (RPMA) indicated that p53 was phosphorylated on two residues, Ser15 and Ser46 upon infection with the virulent strain of RVFV (ZH-501) [18]. This indicates that the p53 pathway may be activated upon infection with RVFV. The p53 tumor suppressor protein is activated in response to genomic stress, such as DNA damage, and has been implicated in governing many cellular processes such as cellular homeostasis, apoptosis, cell cycle arrest, activation of DNA repair, and cellular senescence. p53 can achieve these functions due to its role as a transcription factor, inducing or repressing numerous target genes. p53 can also act in a non-transcriptional manner through direct protein-protein interaction [19]. When bound to murine double minute 2 (MDM2), its negative regulator, p53 is targeted for degradation by the proteosome. However, when genomic stress occurs, several post-translational modifications occur, including heavy phosphorylation of the N-terminus, that help to stabilize p53 and lead to its accumulation. Phosphorylation can be initiated by DNA damage response (DDR) protein kinases such as Ataxia-Telangiectasia Mutated (ATM), ATM and Rad3-related protein (ATR), Checkpoint Kinase 1 (chk1) and Checkpoint Kinase 2 (chk2) [20]. The N-terminus contains the trans-activation domain (TAD), responsible for the transcriptional regulation of the protein, and the C-terminal domain of the protein is important for specific DNA binding of p53 [21]. The phosphorylation at specific sites can control the activity of the protein, either through its accumulation, activity in the TAD, or through modulation of DNA consensus sequences affinity [22]. The serine residues located in the TAD (Ser9, 15, 20, and 37) are important for the transcriptional regulation of the protein. Phosphorylation of Ser15 and Ser37 are also important for the stability of the protein. Ser20 phosphorylation can enhance tetramerization, stability, and activity [23]. Ser46 has been implicated in the induction of apoptosis through the transcriptional regulation of p53AIP1 (p53 regulated apoptosis inducing protein 1) [24]. Phosphorylation of the C-terminal end of the p53 protein is important for sequence specific DNA binding regulation, oligomerization state, nuclear localization and export, as well as ubiqutination of the protein [25]. Phosphorylation at Ser392, located in the C-terminus affects growth arrest, DNA binding and transcriptional activation [26]. The differential response to p53 phosphorylation is responsible, in part, for the multifunctional nature of the protein.

p53 acts as a transcription factor to induce the regulation of multiple genes. In doing so, p53 can control many different cellular responses to stressors such as DNA damage. Transcriptional targets of p53 regulating cell cycle arrest include 14-3-3σ, p21 and Reprimo. p21 regulates the progression of cells in the G1 phase of the cell cycle through binding to and inhibiting the activity of CDK2 and CDK4. Activation of p21 by p53 leads to G1 cell cycle arrest [27]. p53 regulation of DNA damage repair induces the transcriptional targets Growth Arrest and DNA Damage Inducible 45 (GADD45) and Proliferating Cell Nuclear Antigen (PCNA), among others. The apoptotic pathway regulated by p53 is activated through two major pathways, the extrinsic and the intrinsic pathways and through both transcription dependent as well as independent mechanisms [28]. The intrinsic pathway is reliant on the activation of Bcl-2 family members, comprised of both pro-apoptotic (Bid, Bax, Puma and Noxa) and anti-apoptotic proteins (Bcl-Xl), which control the release of cytochrome c from the mitochondria [29]. p53 induces the expression of many of the Bcl-2 pro-apoptotic family members, activating the intrinsic apoptotic mitochondrial pathway and promoting cell death.

The host’s response to a viral pathogen plays an important role in the outcome of the infection. Viruses have been shown to utilize host cellular signaling pathways to facilitate infection and viral replication [30], [31], [32]. We have previously shown that p53 was phosphorylated on Ser15 and Ser46 following infection with RVFV ZH-501 [18]. Here we expand upon that data to characterize additional phosphorylation events on p53. We find that numerous Ser residues are phosphorylated following RVFV infection and that these events are highly dependent on NSs. p53 transcriptional targets were examined and the apoptotic gene, Noxa, was most highly upregulated following RVFV infection. Interestingly, p53 null cells were resistant to RVFV induced cell death and were not as efficient at viral production. These data suggests that RVFV utilizes the p53 pathway to facilitate viral production, possibly through the manipulation of the p53 dependent apoptotic pathway.

## Materials and Methods

### Cell Culture

Human small airway lung epithelial cells (HSAECs) were obtained from Cambrex Inc., Walkersville, MD and maintained in Ham’s F12 medium and supplemented with 1% penicillin/streptomycin, 1% L-glutamine, 1% nonessential amino acids, 1% sodium pyruvate, 0.001% of 55 mM β-mercaptoethanol (Gibco Cat # 2195-023) and 10% Fetal Bovine Serum (FBS). Vero cells (ATCC Cat # CCL-81), MDA-MB-231 human breast cancer cells (ATCC Cat # HTB-26) and MCF-7 human breast adenocarcinoma cells (ATCC Cat # HTB-22) were maintained in Dulbecco’s modified minimum essential medium (DMEM) supplemented with 10% FBS, 1% penicillin/streptomycin and 1% L-glutamine. T47D (ATCC cat # HTB-133) cells were maintained in RPMI medium supplemented with 10% FBS, 1% penicillin/streptomycin and 1% L-glutamine. A549 (ATCC Cat # CCL-185) cells were maintained in Ham’s F12 medium supplemented with 10% FBS, 1% penicillin/streptomycin and 1% L-glutamine. HCT-116 human cancer cells and p53 heterozygous and null derivatives (p53+/+, p53+/− and p53−/−) were maintained in McCoys 5A medium supplemented with 10% FBS, 1% penicillin/streptomycin and 1% L-glutamine. All cell lines were maintained in 5% CO^2^ at 37°C and in accordance with the distributor’s guidelines.

### Viruses

The MP-12 strain of RVFV, a live attenuated virus (LAV) derivative of the ZH548 strain, was isolated from a patient with uncomplicated RVFV infection in 1977. MP-12 was generated by 12 serial passages in MRC5 cells in the presence of 5-fluorouracil, which induced a total of 25 nucleotide changes across the three viral genome segments [33]. arMP-12-del21/384 (herein referred to as arMP-12 ΔNSm) has a large deletion in the pre-Gn region of the M segment and as a result does not express NSm, 78 kDa, 75 kDa, or 73 kDa proteins encoded by this region [9]. rMP-12-NSdel (herein referred to as rMP-12 ΔNSs) completely lacks the NSs ORF [34]. rMP-12 that has a C-terminal Flag-tagged NSs inserted in place of the NSs ORF is referred to as rMP-12-NSs-Flag [35].

### Viral Infection

For experiments using RVFV MP-12 strain, cells were cultured at a density of 1×10^6^ per well in 6-well plates. Cultured cells were infected with MP-12 (attenuated strain of ZH548), rMP-12ΔNSs or arMP-12ΔNSm viruses at the specified multiplicity of infection (MOI). Cells were infected by overlaying a 400 µl suspension of viral media on the cells and incubating them for 1 hour at 37°C at 5% CO_2_. Following the 1 hour incubation, the viral media was removed and the cells were washed with phosphate buffered saline (PBS) without Mg and Ca and replaced with 2 ml of cell specific media. For cell viability assay infections, cells were plated at 2.5×10^4^ in a 96 well plate. The same procedure as described above was used except a 25 µl suspension of viral media is used and 200 µl of cell specific media is replaced after 1 hour incubation and washing. Cells were maintained at 37°C at 5% CO_2_ until appropriate collection time.

### Western Blot Analysis

Cells were collected in lysis buffer: 1∶1 mixture of T-PER reagent (Pierce, IL), 2× Tris-glycine SDS sample buffer (Novex, Invitrogen), 33 mM DTT, and protease and phosphatase inhibitor cocktail (1× Halt cocktail, Pierce)] and boiled for 10 min. Twenty-five µl of cell lysates were separated on NuPAGE 4–12% Bis-Tris gels (Invitrogen) and transferred to nitrocellulose or PVDF membranes using the iBlot Gel Transfer apparatus (Invitrogen) or either an overnight wet transfer at 70 mA or a 2 hr wet transfer at 250 mA at 4°C. The membranes were blocked with previously boiled 3% dry nonfat milk solution in 1× PBS +0.1% Tween (PBS-T) for 1 hour at either room temperature or 4°C. The primary antibodies were diluted in 3% milk solution at a 1∶1000 dilution (with the exception of β-actin, 1∶10,000 dilution) and incubated overnight at 4°C. The membranes were then washed 3 times with PBS-T and incubated with secondary HRP-coupled goat anti-rabbit and anti-mouse antibody diluted 1∶10,000 in 3% milk for 2 hours and then washed 5 times with PBS-T, 10 min washes. The primary antibodies used were: anti-p53 (cat #9282, Cell Signaling), anti-phospho-p53 Antibody Sampler Kit (Cat #9919, Cell Signaling), HRP conjugated β-actin (Cat# ab49900-100, Abcam), and anti-RVFV N (kind gift from Dr. Connie Schmaljohn, USAMRIID). The western blots were visualized by chemiluminescence using SuperSignal West Femto Maximum Sensitivity Substrate kit (ThermoScientific) and a Bio Rad Molecular Imager ChemiDoc XRS system (Bio-Rad).

### Creation of Nuclear and Cytoplasmic Extracts

One million cells were resupended in 80 µl of Buffer A (10 mM KCl, 10 mM MgCl2, 10 mM HEPES, 1 mM EDTA, 1 mM DTT, 0.1% PMSF and EDTA-free complete protease inhibitor cocktail) with 0.5% NP-40, and incubated for 10 minutes on ice. Nuclei were centrifuged at 5,000 g for 5 minutes and the supernatant saved as the cytoplasmic extract (CE) for further analysis. Next, the nuclei were washed one time with 200 µl of Buffer A with 0.5% NP-40 and the centrifugation step repeated. The nuclei were then resuspended in 80 µl of Buffer B (450 mM NaCl, 1.5 mM MgCl2, 20 mM HEPES, 0.5 mM EDTA, 1 mM DTT, 0.1% PMSF and EDTA-free complete protease inhibitor cocktail) and incubated on ice for 10 minutes. Finally, lysates were centrifuged at 20,000 g for 10 minutes and the nuclear extract found in the supernatant saved for further analysis.

### Immunofluorescent Staining

HSAEC’s cells were grown on coverslips in a 6-well plate, infected with MP-12 as described above and washed with ice cold PBS (without Ca and Mg) then fixed with 4% formaldehyde. Cells where permeabilized with 0.5% Triton X-100 in PBS for 20 minutes and then washed 2 times in PBS. The cells were then blocked for 10 minutes at room temperature in 3% BSA in PBS. Primary antibodies anti-p53 (Cat# 9282, Cell Signaling), anti-RVFV N protein, anti-Flag (Sigma) diluted 1∶1000, was incubated in fresh blocking buffer at 37°C for 1 hour and washed 3 times for 3 min in 300 mM NaCl with 0.1% Triton X-100. Alexa Fluor 568 anti rabbit (Cat# A10042 Invitrogen) and Alexa Fluor 488 anti mouse (Cat# A11001 Invitrogen) dilution 1∶200, were used as a secondary antibodies and treated in the same manner as the primary antibody. DAPI, dilution 1∶1000, was used to visualize nuclei. Fluorescence microscopy was carried out using a Nikon Eclipse 90i microscope.

### Cell Viability Assay

Cells were cultured and infected as previously described at the indicated MOI. At the appropriate time point a cell viability assay using CellTiter-Glo Cell Luminescent Viability Assay (Promega) was performed according to manufacturer’s protocol. Briefly, an equal volume of room temperature media and CellTiter-Glo reagent was added to the cells. The plate was shaken for 2 min on an orbital shaker and after a 10 minute room temperature incubation; the luminescence was detected using the DTX 880 multimode detector (Beckman Coulter).

### Plaque Assay

Supernatants were collected from the p53+/+ and p53−/− cell viability experiment at the 96 hr time point from the MOI of 0.1 and 5.0 samples and stored at −80°C. Vero cells were plated in 6 well plates at 1×10^6^ cells per well. When cells reached 90% to 100% confluency, they were infected as follows in duplicates for each dilution. Viral supernatants are diluted 1∶10 in complete DMEM media from 10^−1^ to 10^−8^. Four hundred µl of each viral dilution was added to the cells. After the one hour infection an overlay of 3 ml of a 1∶1 solution of 0.5% agarose in diH_2_0 with 2× EMEM for plaque assays, containing 5% FBS, 1% L-Glutamine, 2% penicillin/streptomycin, 1% nonessential amino acids, and 1% sodium pyruvate was added to each well, allowed to solidify and incubated at 37°C at 5% CO_2_ for 72 hrs. After 72 hrs, cells were fixed using 4% formaldehyde for 1 hr at room temperature. The agar plugs were then discarded and fixed cellular monolayers were stained with a 1% crystal violet, 20% methanol solution for 15 min, visualizing plaques. Averages were taken from duplicates, with dilutions containing fewer than 5 or more than 100 plaques being discounted. The viral titer was calculated as follows: pfu/ml  =  average of 2 plaque counts ×2.5 (dilution factor) × dilution.

### Quantitative RT-PCR

HSAEC cells were grown in 6 well plates and infected with MP-12 at an MOI 3.0 as described above. The cells were harvested at 24 and 48 hours P.I. in 350 µl of Buffer RLT + β-mercaptoethanol. The RNA was extracted using RNeasy Mini Kit (Qiagen) in accordance with manufacturer’s protocol. The RNA was DNAse treated (DNase I-RNase-Free, Ambion) to remove any contaminating DNA. Two hundred ng of total RNA was used in a twenty µl cDNA reaction using the iScript Select cDNA Synthesis kit (BIO-RAD) in accordance with manufacturer’s protocol. For q-PCR, the template cDNA was added to a 20 µl reaction with SYBR® GREEN PCR master mix (Applied Biosystems) and 0.2 µM of primer. Refer to Table 1 for primer sequences. cDNA was amplified (1 cycle −95°C for 10 min, 40 cycles- 95°C for 15 sec and 60°C for 1 min) using the ABI Prism 7000. Fold changes were calculated relative to Actin using the ΔΔCt method.

**Table 1:** Primer Sequences.

Primer Pair	Forward Sequence 5′-3′	Reverse Sequence 5′-3′
Bax	TGC TTC AGG GTT TCA TCC AG	CGC GGC AAT CAT CCT CTG
Puma	GGG CCC AGA CTG TGA ATC CT	ACT TGC TCT CTC TAA ACC TAT GCA
Noxa	GTG CCC TGG GAA ACG GAA GA	CCA GCC GCC CAG TCT AAT CA
p21	CTG GAG ACT CTC AGG GCG AAA	GAT TAG GGC TTC CTC TTG GAG AA
GADD45	TGC TCA GCA AAG CCC TGA GT	GCT TGG CCG CTT CGT ACA
14-3-3σ	GGC CAT GGA CAT CAG CAA GAA	CGA AAG TGG TCT TGG CCA GAG
MDM2	GTG AAT CTA CAG GGA CGC CAT C	CTG ATC CAA CCA ATC ACC TGA A
p62	TGC AGG CAC AAC TAA CTT	ACT TGA CTC ACA ACA AGG TCT TT
Actin	GCC GGT CGC AAT GGA ACA AGA	CAT GGC CGG GGT GTT GAA GGT

## Results

### p53 is Phosphorylated on Several Residues Following Infection with RVFV

**Figure 1:**
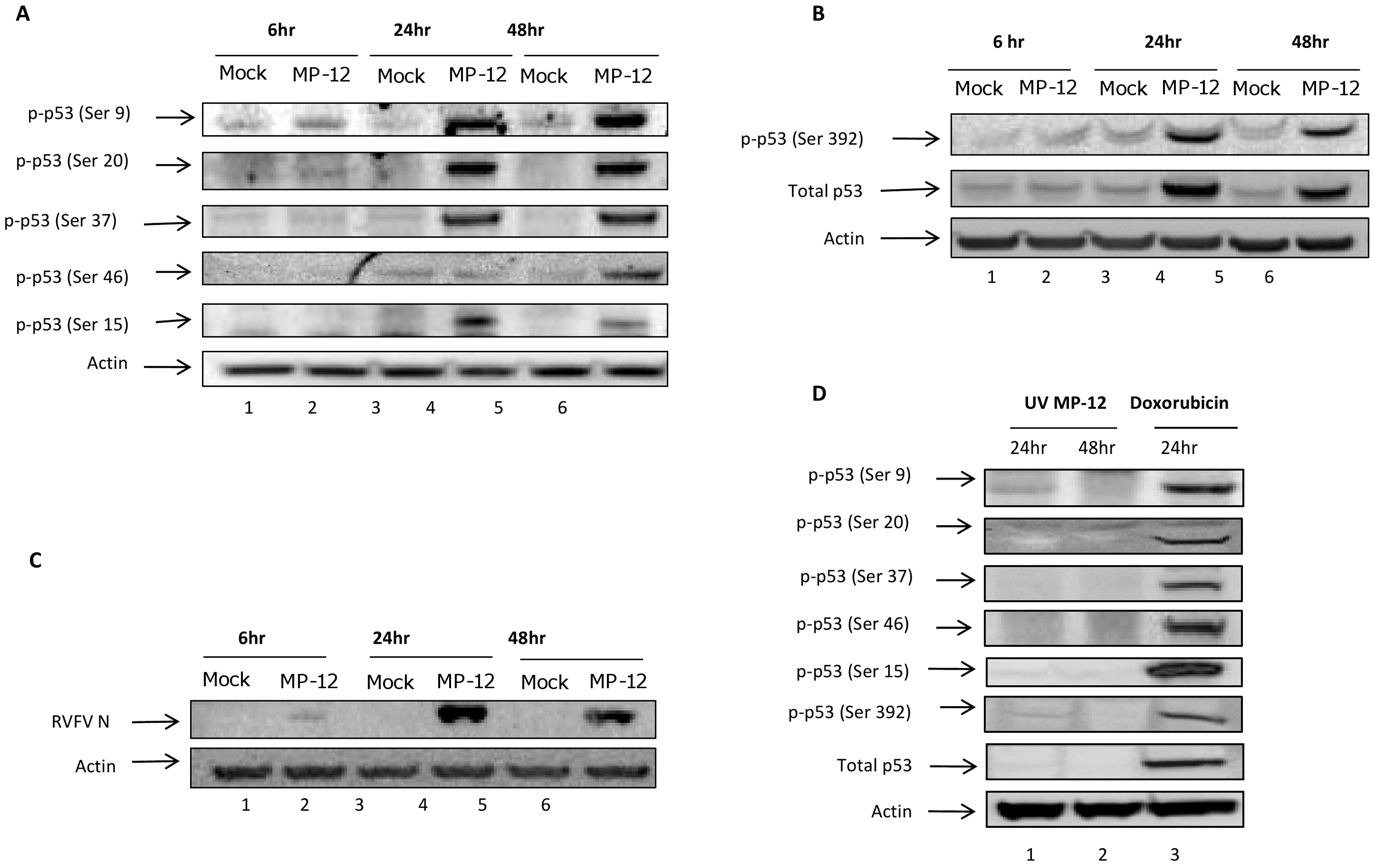
p53 is phosphorylated on several residues following infection with RVFV. A) HSAECs were either mock or MP-12 infected at an MOI of 3.0. Cells were collected 6, 24 and 48 hours post infection and lysates were analyzed by western blotting for antibodies against p53 at the residues indicated (Ser 9, Ser 20, Ser 37, Ser 46 and Ser 15). B) HSAECs infected in the same manner as Fig. 1A were collected at the time points indicated and analyzed by western blotting for antibodies against total p53 and p53 (Ser392). C) HSAECs were treated in the same manner as Fig. 1A and Fig. 1B and analyzed by western blotting for antibodies against the N protein of RVFV. D) HSAECs were infected with UV inactivated MP-12 (MOI 3.0) and collected at 24 and 48 hours post-infection. In parallel, HSAECs were treated with doxorubicin (1 µM) and collected at 24 hours. Cell lysates were analyzed by western blot analysis as describe in panels A and B. Actin is used as a loading control in all panels.

Phosphorylation plays a role in regulating many signaling pathways. In response to DNA damage and stress stimuli, a series of phosphorylation events take place, mainly on the N-terminus, that contributes to the activation of p53 [25]. In addition, there are a series of other specific serine sites of p53 within the TAD that have been shown to regulate the activation of p53 in a specific manner [36]. Previous studies have shown that p53 Ser15 phosphorylation is up-regulated upon infection with the RVFV ZH-501 strain at low MOIs [18]. We were interested in determining if additional p53 serine residues were phosphorylated following RVFV infection. First, we assessed the status of p53 Ser15 and Ser46 phosphorylation following infection with the live attenuated vaccine strain of RVFV, MP-12. Results in Figure 1A indicate that both residues were phosphorylated following MP-12 infection, indicating that the attenuated virus was performing similarly to the virulent virus. In addition, our data indicated that RVFV MP-12 infection induces phosphorylation of all p53 serine sites examined. Residues Ser9, Ser20, Ser37, Ser15 were highly phosphorylated beginning at 24 hours, whereas Ser46 was not highly phosphorylated until 48 hours (Figure 1A). p53 Ser46 has been implicated in the induction of apoptosis [24]; therefore the delayed response could contribute to MP-12 induced apoptosis. p53 Ser392, a residue known to contribute to both transcription activation and DNA binding [26], was also phosphorylated in a manner similar to that seen with N-terminal residues (Figure 1B). Upon activation, reduced interaction between p53 and its negative regulator MDM2 leads to accumulation of p53 where it is then stabilized [37]. In agreement with this model, total p53 accumulation is apparent 24 and 48 hours post infection (Figure 1B), coinciding with the phosphorylation pattern seen at multiple serine sites of p53. RVFV nucleoprotein (N) expression was also monitored following infection (Figure 1C). As controls, the phosphorylation of p53 was assessed following infection with UV inactivated MP-12 and following doxorubicin treatment (Figure 1D). Doxorubicin treatment resulted in the phosphorylation of p53 on all residues examined. In contrast, we did not observed phosphorylation or stabilization of p53 following infection with UV inactivated MP-12. Collectively, these results demonstrate that in response to RVFV infection, p53 is phosphorylated on multiple serine residues, both in the N-terminal and C-terminal.

### p53 Localizes to the Nucleus Following Infection with RVFV

**Figure 2:**
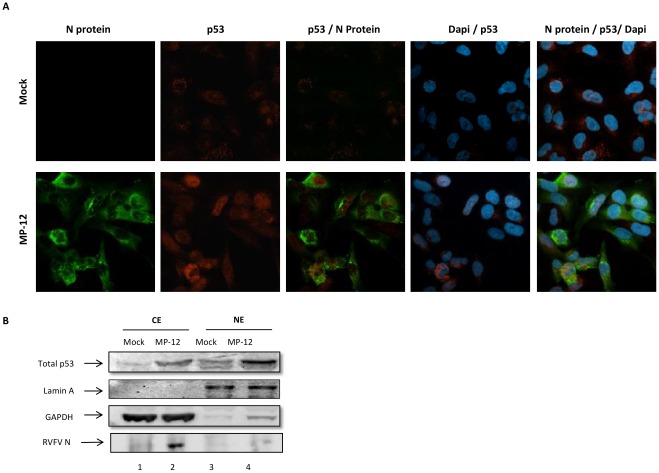
p53 localizes to the nucleus following infection with RVFV. A) HSAECs were either mock infected or infected with MP-12 (MOI 3.0). At 24 hours post infection the cells were washed in PBS without Ca and Mg, permeabilized with Triton X-100 and immunostained with anti-p53 primary antibody using Alexa-Fluor 568 secondary antibody and with anti-RVFV N protein antibody using Alexa-Fluor 488 secondary antibody. The nucleus was stained with DAPI. B) HSAECs were mock infected or infected with MP-12 (MOI 3.0) and collected at 24 hours post-infection for the creation of cytoplasmic and nuclear extracts, CE and NE. Western blot analysis was performed with antibodies against lamin, GAPDH, total p53 and RVFV N protein.

In normal unstressed cells p53 is present in low amounts. This is due in part to its ubiquitination at the C-terminal end by its negative regulator MDM2 and its export from the nucleus to the cytoplasm where it is degraded by the proteosome [38]. When cells are stressed, p53 accumulates in the nucleus where it can regulate its transcriptional targets [38] to induce events such as apoptosis and cell cycle arrest. To visualize the distribution of p53 upon infection with RVFV, HSAECs were either mock infected or infected with MP-12 (MOI 3.0) and collected 24 hours later. p53 was visualized after the cells were immunostained with anti-p53 and Alexa fluor 568 secondary antibody (red) via confocal microscopy. The N protein of RVFV was visualized after staining the cells with anti-N RVFV protein and Alexa fluor 488 secondary antibodies (green). The nucleus was visualized by DAPI staining (blue). Figure 2 shows that in uninfected cells, p53 expression is low and appears evenly distributed throughout the cytoplasm with only basal levels seen in the nucleus. Upon infection with MP-12 (visualized with the RVFV N protein), p53 expression is increased overall and p53 localizes within the nucleus with slight distribution within the cytoplasm. Interestingly, the nuclear p53 correlated with RVFV infection, as detected by RVFV N. To confirm these results, nuclear and cytoplasmic extracts were analyzed for the expression of p53 in mock or MP-12 infected cells (Figure 2B). Lamin and GAPDH western blot analysis confirmed the purity of the fractions. Following MP-12 infection, p53 was found predominantly in the nucleus (compare lanes 2 and 4). These results show that MP-12 infection induces p53 nuclear localization, presumably where it can regulate its transcriptional targets.

### p53 Phosphorylation is Highly Dependent Upon the Viral Protein NSs

**Figure 3:**
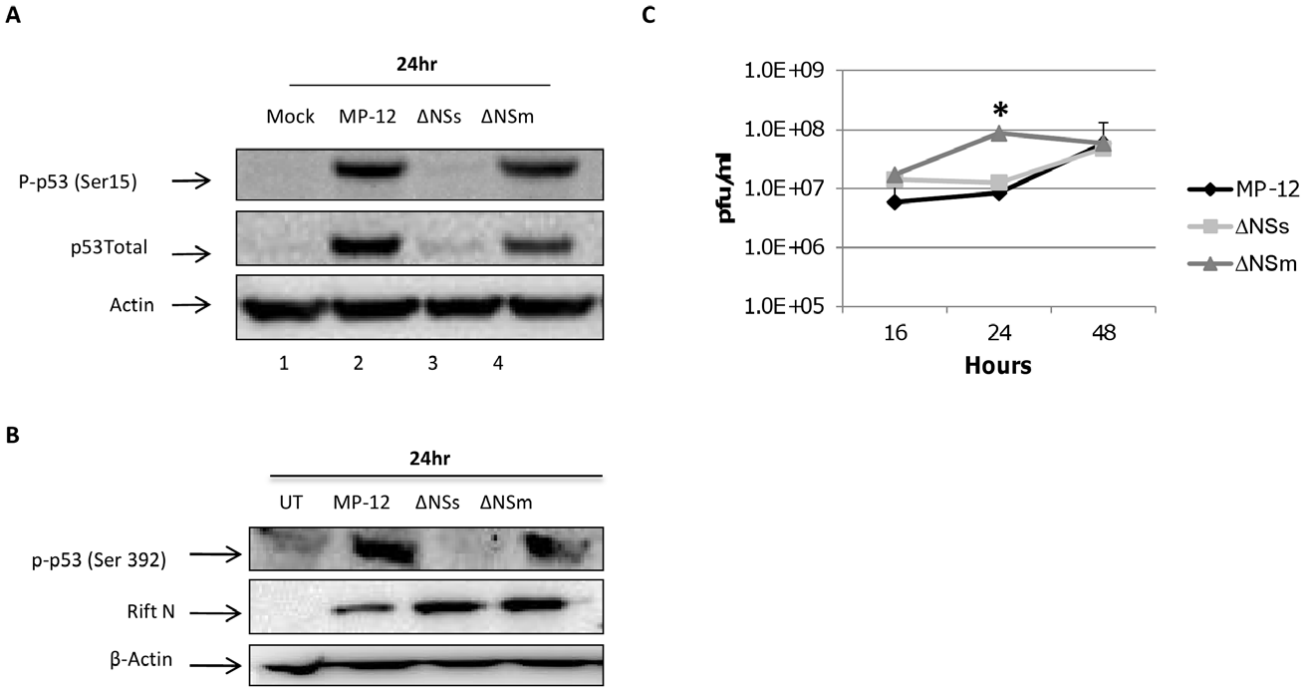
p53 phosphorylation following RVFV infection is NSs dependent. A) Vero cells were either mock infected or infected with MP-12, rMP-12 ΔNSs or arMP-12 ΔNSm viruses at an MOI of 3.0. Cells were collected 24 hours post infection and western blot analysis was performed on the cell lysates using antibodies against p53 (Ser15) and total p53. B) Cells were infected and processed as described in panel A. Western blot analysis was performed for p53 (Ser20), and the N protein of RVFV. Actin was used as a loading control. C) Vero cells were infected with MP-12, rMP-12 ΔNSs or arMP-12 ΔNSm viruses at an MOI of 3.0. Viral supernatants were collected at 16, 24 and 48 hours post-infection and released virus determined by plaque assays. (*) indicates statistically significant difference (unpaired t-test) p<0.001. Error bars indicate standard deviation.

Both the NSs and the NSm proteins of RVFV have been described as virulence factors [12]. The NSs protein forms filaments within the nucleus of infected cells and filament formation is implicated in pathogenesis [39]. In addition, the NSs protein has been shown to suppress the host’s immune response [13], [15], [40]. The NSm protein has been described as having anti-apoptotic functions by blocking the caspase pathway [17]. Because of the importance of these viral proteins in the virulence of RVFV, we wanted to determine whether they were involved in the p53 phosphorylation events observed in Figure 1. To this end we utilized two different viruses generated through reverse genetics. arMP-12-del21/384 (herein referred to as arMP-12 ΔNSm) has a large deletion in the pre-Gn region of the M segment and as a result does not express NSm, 78 kDa, 75 kDa, or 73 kDa proteins encoded by this region [9]. rMP-12-NSdel (herein referred to as rMP-12 ΔNSs) completely lacks the NSs ORF [34]. Vero cells were collected 24 hours after mock infection or infection with MP-12, rMP-12 ΔNSs or arMP-12 ΔNSm. Western blot analysis using anti-p53 total and anti-p53 Ser15 antibodies (Figure 3A) show a very similar pattern of up-regulation with MP-12 and arMP-12 ΔNSm infected cells. Ser392 phosphorylation of p53 was also increased in MP-12 and arMP-12 ΔNSm infected cells at 24 hours (Figure 3B). There is a slight reduction of p53 phosphorylation and p53 accumulation in the arMP-12 ΔNSm infected cells compared to the MP-12 infected cells. As NSm is known to be anti-apoptotic, a loss of p53 signaling in arMP-12 ΔNSm infected cells seems somewhat surprising. However, the mutant virus utilized also lacks the 78 kDa, 75, kDa, and 73 kDa proteins which have unknown functions to date [9]. Specifically, the 78 kDa protein was unable to suppress apoptosis, indicating that it has a function distinct from the anti-apoptotic role of NSm [9]. These data suggest that the loss of one or more of these proteins may have an influence on p53 signaling. In contrast, cells infected with the rMP-12 ΔNSs strain, show a dramatic lack of Ser 15 and Ser 392 phosphorylation and p53 accumulation at 24 hour. As controls for viral replication and protein expression, western blot analysis for the N protein was performed (Figure 3B) and the amount of released virus at 16, 24, and 48 hours post-infection was analyzed (Figure 3C). MP-12 and rMP-12 ΔNSs replicated at similar levels at all time points examined, however arMP-12 ΔNSm displayed an increase in released virus as compared to MP-12 and rMP-12 ΔNSs at 24 hours post-infection. These results demonstrate a strong dependence on the NSs protein for both the rise in p53 levels as well as the phosphorylation events observed upon infection.

**Figure 4:**
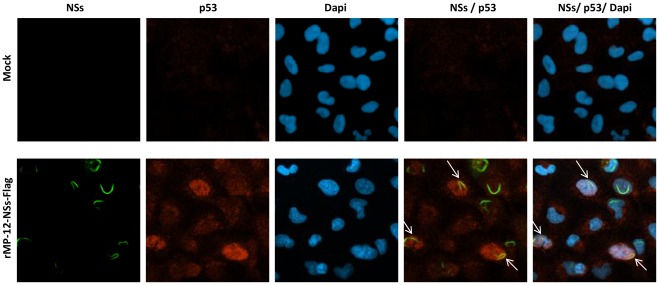
p53 partial co-localizes with RVFV NSs. A) HSAECs were either mock infected or infected with rMP-12-NSs-Flag (MOI 3.0). At 24 hours post infection the cells were washed in PBS without Ca and Mg, permeabilized with Triton X-100 and immunostained with anti-p53 primary antibody using Alexa-Fluor 568 secondary antibody and anti-Flag protein antibody using Alexa-Fluor 488 secondary antibody. The nucleus was stained with DAPI. Arrows indicate NSs Filaments and areas of p53/NSs colocalization.

As p53 phosphorylation was dependent on NSs, we sought to determine if NSs and p53 colocalized following RVFV infection. To this end, HSAECs were either mock infected or infected with MP-12 that has C-terminal Flag-tagged NSs inserted in place of the NSs ORF (rMP-12-NSs-Flag) [35]. Cells were collected 24 hours post-infection and p53 and NSs visualized through confocal microscopy. Figure 4 demonstrates that following rMP-12-NSs-Flag infection, p53 expression is increased and highly nuclear in nature, confirming the results presented in Figures 1, 2 and 3. NSs filaments were observed in approximately 60% of all cells. Cells with NSs filaments displayed nuclear abnormalities, such as lobulated nuclei, as has recently been published by Mansuroglu et al., 2010 [41]. p53 and NSs partially co-localize (white arrows) in approximately 20% of cells visualized and this coincided with strong p53 nuclear staining. Collectively, these results demonstrate the increase in p53 phosphorylation and total protein levels are dependent on NSs and that NSs and p53 partially co-localized following RVFV infection.

### RVFV Infection Induces the Up-regulation of p53 Targeted Genes

**Figure 5:**
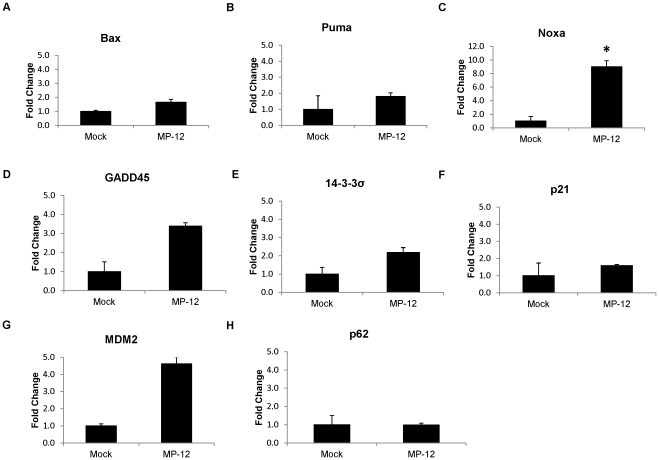
RVFV infection induces the Up-regulation of p53 target genes. HSAECs were either mock infected or infected with MP-12 (MOI 3.0). Cells were collected at 24 hours post infection. RNA was extracted using Qiagen’s RNeasy Mini Kit. After cDNA synthesis, qRT-PCR was performed on the samples using the primers shown (Bax, Puma, Noxa, GADD45, 14-3-3σ, p21, MDM2 and p62) (Panels A–H). Actin was used to normalize the samples. (*) indicates statistically significant difference (unpaired t-test of triplicates) p<0.05. Error bars indicate standard deviation.

Given the function of p53 as a transcription factor, the question arises as to whether RVFV infection results in increased p53 binding to various promoters and regulation of their transcription. To determine if RVFV infection affects the induction of p53 transcriptional targets, quantitative RT-PCR was performed on p53 regulated genes. The p53 target genes involved in apoptosis that were analyzed are Bax, Puma and Noxa (Figure 5A–C). The genes of interest known to be involved in cell cycle control that were analyzed were GADD45, 14-3-3σ, and p21 (Figure 5D–F). In addition, the negative regulator of p53, MDM2 was analyzed (Figure 5G). The results show that in response to RVFV infection a number of p53 target genes were up-regulated (Figure 5A–G). Of the apoptotic genes, Noxa displayed the greatest fold induction (almost 10 fold) as compared to the mock infected cells. Neither Puma nor Bax demonstrated changes greater than 2 fold. 14-3-3σ and GADD45 were both increased by greater than 2 fold; however, p21 transcriptional levels were not significantly altered. MDM2 displayed an increase of almost 5 fold, further supporting the observed p53 stabilization and accumulation following RVFV infection. p62 expression was used as a control for transcription since RVFV infection was recently found to induce p62 downregulation post-transcriptionally [42]. As has been previously reported, p62 transcription was not altered following RVFV infection (Figure 5H). These results indicate that transcriptional targets of p53 involved in the apoptotic pathway and cell cycle control are increased following RVFV infection, with Noxa being significantly up-regulated.

### Loss of p53 Results in Decreased RVFV Induced Death

Having characterized p53 as being activated following infection with RVFV and Noxa as the most significantly up-regulated p53 target, we next sought to determine the influence of p53 on RVFV induced cell death. For this, we utilized two p53 wild-type (WT), A549 and MCF-7, and two p53 mutant (mt), MDA-MB-231 and T47D, cell lines. The mt cell lines are breast cancer cells which have high levels of mutated p53, mutated in the DNA binding region. Cells were mock infected or infected with MP-12 (MOI 0.1 and 1.0) and cell viability assessed at 48 hours and 72 hours post infection. At 48 hours post infection there was little to no loss of viability in the p53 mt cells, whereas the p53 WT cells displayed an MOI dependent loss in viability (Figure S1A). By 72 hours, p53 mt cells began to display a decrease in cell viability, but still to a lesser extent than that of the p53 WT cells (Figure S1B). These results demonstrate that p53 WT status results in a greater loss of cell viability following RVFV infection.

**Figure 6:**
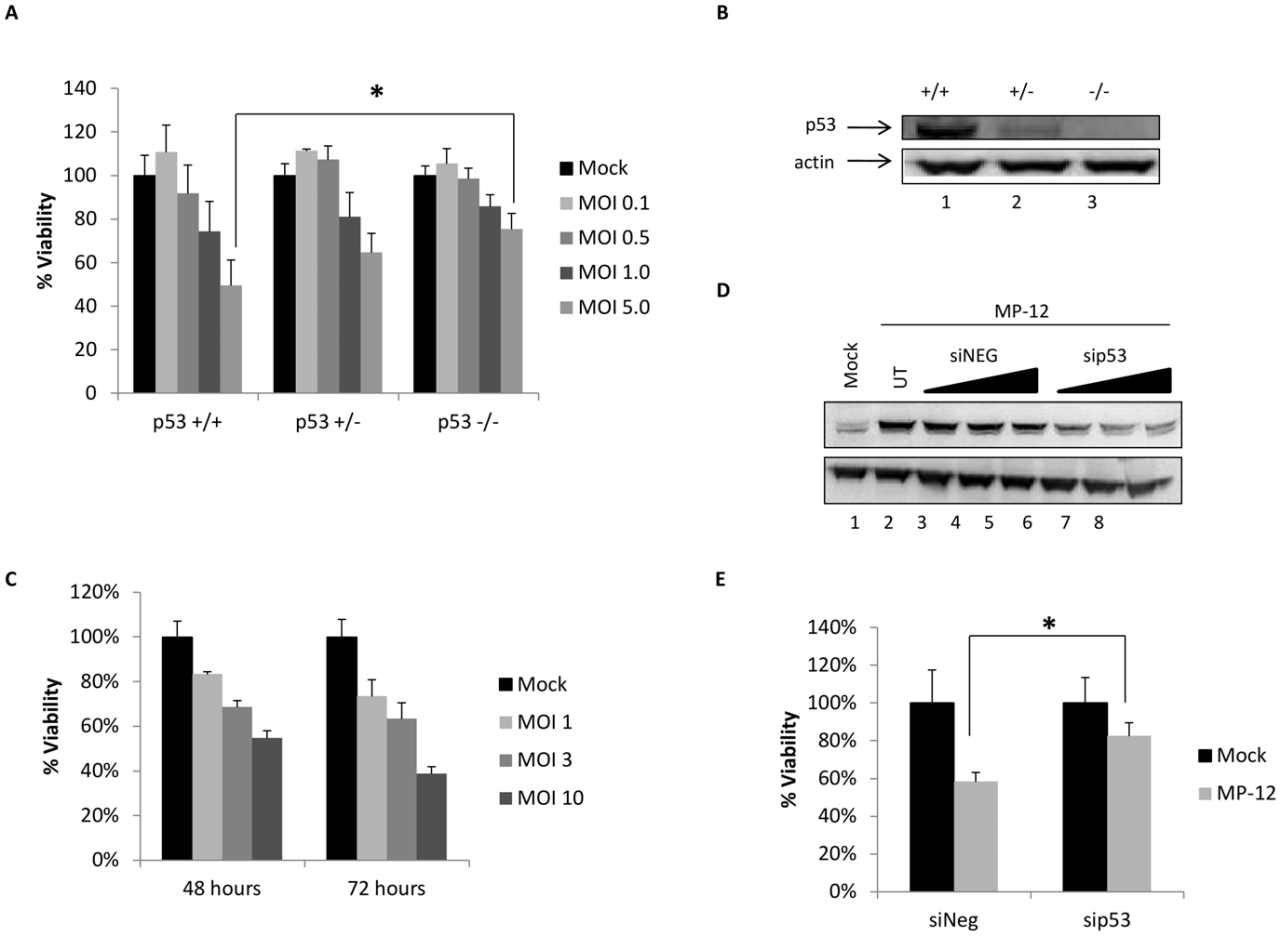
Loss of p53 provides resistance to RVFV induced cell death. A) HCT-116 p53+/+, +/−, and −/− cells were mock infected or infected with MP-12 (MOI 0.1, 0.5, 1.0, and 5.0). Cell viability was determined 96 hours post-infection by CellTiter-Glo Assay (Promega). Viability of the infected cells was calculated relative to the mock infected cells (100%) (average of triplicates shown). (*) Indicates statistically significant difference (unpaired t-test of triplicates) p<0.05. Error bars indicate standard deviation. B) Western blot analysis of uninfected whole cell lysates from HCT-116 p53+/+, +/−, and −/− cells probed with anti-p53 total and actin antibodies. C) HSAECs were either mock infected or infected with MP-12 (MOI 1, 3, or 10). Cell viability was determined 48 and 72 hours post-infection by CellTiter-Glo Assay (Promega). Viability of the infected cells was calculated relative to the mock infected cells (100%) (average of triplicates shown). D) HSAECs were transfected with negative control siRNA (siNEG) or siRNA targeting p53 (sip53) at 50, 100, or 200 nM using attractene’s fast-forward method. Twenty-four hours post-transfection, cells were infected with MP-12 and collected 24 hours post-infection. Cell lysates were analyzed by western blot analysis for total p53 and actin. Lane 1 is a mock infected and untransfected control. E) HSAECs were transfected with siNEG or sip53 (100 nM) by attractene’s fast-forward method. Twenty-four hours post-transfection cells were mock infected or infected with MP-12 (MOI 10). Cell viability was determined 72 hours post-infection by CellTiter-Glo Assay (Promega). Viability of the infected cells was calculated relative to the mock infected cells (100%) (average of triplicates shown). (*) Indicates statistically significant difference (unpaired t-test of triplicates) p<0.01. Error bars indicate standard deviation.

To further characterize p53 as having a role in decreased cell viability following infection by RVFV, we obtained HCT-116 cells completely devoid of p53 (p53−/−, null) as well as the WT (p53+/+) and heterozygous (p53+/−) counterparts. Cell viability assays were performed on each of the cell lines 96 hours after mock infection or infection with MP-12 at the indicated MOIs. The results are analogous to those obtained for the mutant cells lines. Particularly at the higher MOIs (MOI 0.5–5.0), the p53 null cells showed increased survival compared to the WT cell line after RVFV challenge (Figure 6A). Interestingly, the heterozygous cell line (p53+/−) show an intermediate increase in cell viability under the same conditions. Figure 6B confirms through western blot analysis that the expression of p53 in the p53 null cells is absent, while the heterozygous cells show less p53 protein than the WT p53 cells. It is interesting to note that the HCT-116 cells appear to be inherently resistant to RVFV induced cell death, as at 96 hours post-infection, 50% viability remained. Confocal microscopy imaging of RVFV N protein indicated that only around 10% of all cells were infected at 96 hours (data not shown). It is likely that by 96 hours most of the infected cells have undergone apoptosis and the cells that remain are largely uninfected.

We sought to confirm these findings in a cell line that is more susceptible to RVFV induced cell death. To this end, HSAECs were infected with MP-12 at various MOIs (1, 3, 10) and cell viability accessed at 48 and 72 hours post-infection (Figure 6C). At 72 hours and a MOI of 10, only 39% of cells remained viable. Therefore, this time point was chosen to examine the influence of p53 siRNA mediated knockdown on RVFV induced cell death. A titration of negative control siRNA (siNEG) or p53 siRNA (sip53) was performed to determine the optimal concentration of siRNA to be utilized. All three concentrations of siRNA (50, 100, 200 nM) resulted in a loss of p53 protein expression (Figure 6D). The mid concentration (100 nM) was chosen for additional experiments as it was effective at reducing p53 protein levels and the higher concentration of 200 nM did not reduce p53 levels further at 48 hours post-transfection. Next, cell viability was assessed 72 hours following MP-12 infection in cells that were transfected with siNEG or sip53. Loss of p53 through siRNA knockdown resulted in significantly less cell death following RVFV infection (Figure 6E). These results demonstrate a direct correlation between loss of p53 function and a greater resistance to RVFV induced cell death, suggesting that p53 plays a role in RVFV induced apoptosis.

### RVFV Production is Decreased in p53 Null Cells

**Figure 7:**
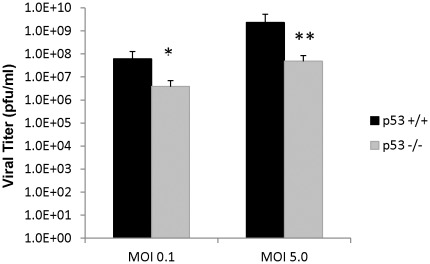
RVFV production is decreased in p53 null cells. HCT-116 p53+/+ and −/− cells were mock infected or infected at the indicated MOIs (0.1 and 5.0). At 96 hours post infection viral supernatants were collected and cells were used in a Cell Viability Assay (Figure 4b). Plaque assays were performed in triplicates using the supernatants and the average viral titers (pfu/ml) of the triplicates are shown. (*) Indicates statistically significant difference (unpaired t-test of triplicates) p<0.05, or (**) p<0.01. Error bars indicate standard deviation.

Next, we assessed the amount of viral production in the presence or absence of p53. Plaque assays were performed using viral supernatants collected at 96 hours from the p53 WT (p53+/+) and p53 null (p53−/−) cells infected with MP-12 (MOI 0.1 and 5.0). The p53 null cells showed an average viral titer of 3.9×10^6^ pfu/ml at an MOI of 0.1 (Figure 7). This is decreased more than a log as compared to the viral titers of 6.1×10^7^ pfu/ml obtained from the p53 WT cells. At the higher MOI (5.0), the decrease in viral titer was more dramatic in the p53 null cells with nearly a two log difference compared to the p53 WT cells. These results demonstrate that lack of p53 results in less RVFV being produced.

## Discussion

Like all viruses, RVFV employs the host’s cellular machinery to complete its lifecycle. Identifying particular host cellular networks that aid in viral propagation is important for further understanding of the viral lifecycle as well as in the development of novel therapeutics to combat infection. Given the importance of p53 in the regulation of many cellular processes such as growth arrest, DNA damage repair, and apoptosis, it would seem logical that RVFV would develop mechanisms to utilize components of these pathways for its benefit.

The activation of the p53 pathway following RVFV infection is evident in the consistently up-regulated phosphorylation observed at every serine phosphorylation site examined, both in the TAD and the C-terminal domain. This universal phosphorylation is interesting given the specific functions each residue seems to exhibit. As previously mentioned, Ser15 and Ser20 are important in the activation and stability of p53, while Ser46 plays a role in apoptosis. Ser392, located in the C-terminal domain, is important for DNA binding and transcriptional activation and is also highly phosphorylated. In addition to the individuality of each residue’s function, is the fact that these residues can be phosphorylated by several different kinases [36]. Recent work from our laboratory indicates that RVFV infection causes opposing activation of ATM and ATR pathways, where ATM and chk2 phosphorylation are up-regulated at 24 hours post-infection, whereas ATR phosphorylation is down-regulated [43]. These data indicated that following RVFV infection, the p53 pathway may be at least partially activated through an ATM-chk2 specific signaling cascade; however, there are likely additional upstream kinases contributing to the robust p53 phosphorylation. ATM and chk2 activation following RVFV were shown to be responsible for the RVFV induced S phase arrest [43]. Cell cycle arrest can be transient to provide time for the cell to repair itself prior to proceeding into the next phase of the cell cycle or if the insult is considered too great (as in the case of RVFV infection), apoptosis can be initiated.

Although apoptosis can be initiated through other mechanisms, phosphorylation of p53 at Ser46 is considered to be a primary determinant of apoptotic induction through the p53 pathway [44]. Interestingly, phosphorylation of p53 at Ser46 is not up-regulated until 48 hours post infection, when apoptotic signaling occurs in HSAECs [45]. Specifically, we have previously shown that in HSAECs, even at a relatively low MOI (0.002) at 48 hours post infection, RVFV causes an increase in effector caspases 3, 6 and 7 and an increase in the sub G1 population, indicative of apoptosis [18]. In addition, our studies indicate that loss of p53 results in cell being more resistant to RVFV induced cell death. Given the importance of the NSs protein in the virulence of RVFV, it is not surprising that the phosphorylation of at least two residues of p53 (Ser15 and Ser392), as well as total p53 accumulation within the cell, are dependent on NSs. NSs localizes to the nucleus forming large filaments, which are responsible for a high incidence of nuclear anomalies and known chromosome cohesion and segregation defects [41]. Based on these properties, it would be plausible that NSs dependent DNA damage is occurring and contributing to the activation of p53 through the DDR pathway. However, our recently published work indicated that RVFV infection does not induce DNA damage [43], as expected. Our data also demonstrated a partial co-localization of NSs and p53 specifically on NSs filaments. Current studies are investigating the possibility of NSs interacting with p53 directly or as part of a larger protein complex present on NSs filaments. It is also possible that NSs is not interacting with p53, but rather acting in conjunction with other host proteins upstream of p53 to induce p53 phosphorylation and stabilization.

Our quantitative RT-PCR results show a general up-regulation of p53 transcriptional targets upon infection with RVFV. This is interesting considering that the NSs protein of RVFV has been shown to target the general transcription factor TFIIH to suppress host transcription [46]. Specifically, NSs can interact with the p44 subunit of TFIIH at specific sites to form filaments and reduce levels of the p62 subunit of TFIIH [42]. While these proteins are reduced, they are not completely degraded upon infection with RVFV and may allow for transcriptional activation of selected genes. In addition, the p53 TAD interaction with the p62 subunit was shown to be enhanced by phosphorylation at Ser46 of p53 [47]. Given that Ser46 phosphorylation is up-regulated upon infection, perhaps this increased affinity for p62 could compensate for the reduced levels of p62. p53 also interacts with TFIID, another basal transcription factor, providing another potential mechanism for p53 regulated transcriptional activation [48].

The manipulation of the p53 pathway has been demonstrated in numerous viral infections [49]. For chronic cancer causing viruses, such as human papilloma virus (HPV) and human T-cell leukemia virus 1 (HTLV-1), p53 inhibition by viral proteins contributes to transformation [50], [51], [52], [53]. Specifically the HPV E6 protein induces the degradation of p53, while the HTLV-1 Tax protein has multiple proposed mechanisms of p53 inactivation, including sequestration of transcriptional cofactors CREB binding protein (CBP) and p300 [50], [51], [52], [53]. For acute viral infections, p53 inactivation is often times postulated as a means to subvert the anti-viral response and/or as a mechanism to inhibit apoptosis to allow sufficient time for efficient viral replication [49]. However, there are viruses that cause activation of p53 to enhance their replication. For example, West Nile Virus (WNV) capsid protein induces nuclear accumulation of p53 and prevents it from associating with MDM2, resulting in p53 stabilization [54]. In WNV infected cells, the p53 transcriptional target, Bax, was up-regulated and contributed to apoptosis [54]. Interestingly, p53 null cells displayed decreased apoptosis and viral replication, indicating that p53 enhances WNV replication [54]. In addition, viral activation of p53 can lead to efficient viral release and dissemination through promoting apoptosis [55]. Similar to WNV infection, we observed an increase in apoptosis and RVFV production in p53 WT cells. We hypothesize that p53 activation by NSs is important for the induction of apoptosis and viral release at later stages of viral infection. Conversely, RVFV NSm protein serves an anti-apoptotic role, functioning to balance the pro-apoptotic p53 signaling, allowing a delay in apoptosis until efficient viral replication can proceed.

It is important to note that a recent publication by Verbruggen et al. demonstrated a lack of p53 activation by the orthobunyavirus La Crosse [56]. In this study, other markers of the DNA damage response pathway, such as phosphorylation of histone H2A.X, degradation of RNA polymerase II, and transcriptional activation of pax6 were observed. These findings were also dependent on the La Crosse viral protein NSs, which has similar functions to RVFV NSs including transcriptional inhibition and interferon antagonism [56], [57]. La Crosse NSs while being found in the nucleus does not form filaments. RVFV infection also induces histone H2A.X phosphorylation and a number of other classical DNA damage responsive proteins [43]. Therefore, these recent findings indicate a clear distinction between the host signaling induced by La Crosse NSs and RVFV NSs, namely the modulation of the p53 pathway. This is not entirely surprising as the two viruses have evolved distinct mechanism to facilitate interferon antagonism [58]. These data suggest that these viruses may also utilize distinct pathways for the induction of apoptosis, with RVFV cell death being influenced by p53.

The overall conclusions of this research points to a role of the p53 pathway in the induction of RVFV induced apoptosis. Upon infection of RVFV, p53 is activated as supported by the amplified phosphorylation of p53 on multiple sites and the localization of p53 within the nucleus. The lack of p53 results in a greater resistance to RVFV induced cell death as well as a decrease in viral production. RVFV infection corresponds to an increase in both cell cycle regulator genes and pro-apoptotic genes of the intrinsic apoptotic pathway controlled by p53. Further research is needed to uncover the exact mechanism by which RVFV utilizes the p53 pathway for efficient viral production.

## Supporting Information

Figure S1 **p53 mutant cells are more resistant to RVFV induced cell death.** p53 WT (A549 and MCF-7) and p53 mt (MDA-MB-231 and T47D) cells were plated at 25,000 cells per well in a 96 well plate. Cells were mock infected or infected with MP-12 (MOI 0.1 and 1.0). Cell viability was determined at A) 48 or B) 72 hours post-infection by CellTiter Glo Assay (Promega). Viability of the infected cells was calculated relative to the mock infected cells (100%) (average of triplicates shown). (*) Indicates statistically significant difference (unpaired t-test of triplicates) p<0.01. Error bars indicate standard deviation.
(TIF)

## References

[1] Bouloy M, Weber F (2010). Molecular biology of rift valley Fever virus.. Open Virol J.

[2] Pepin M, Bouloy M, Bird BH, Kemp A, Paweska J (2010). Rift Valley fever virus(Bunyaviridae: Phlebovirus): an update on pathogenesis, molecular epidemiology, vectors, diagnostics and prevention.. Vet Res.

[3] Bird BH, Ksiazek TG, Nichol ST, Maclachlan NJ (2009). Rift Valley fever virus.. J Am Vet Med Assoc.

[4] Ikegami T, Makino S (2011). The Pathogenesis of Rift Valley Fever.. Viruses.

[5] Daubney R, Hudson JR, Garnham PC (1931). Enzootic hepatitis or rift valley fever. An undescribed virus disease of sheep cattle and man from east africa.. The Journal of Pathology and Bacteriology.

[6] Pepin M, Bouloy M, Bird BH, Kemp A, Paweska J (2010). Rift Valley fever virus (Bunyaviridae: Phlebovirus): an update on pathogenesis, molecular epidemiology, vectors, diagnostics and prevention.. Veterinary Research.

[7] Zamoto-Niikura A, Terasaki K, Ikegami T, Peters CJ, Makino S (2009). Rift valley fever virus L protein forms a biologically active oligomer.. Journal of Virology.

[8] Won S, Ikegami T, Peters CJ, Makino S (2006). NSm and 78-kilodalton proteins of Rift Valley fever virus are nonessential for viral replication in cell culture.. J Virol.

[9] Won S, Ikegami T, Peters CJ, Makino S (2007). NSm protein of Rift Valley fever virus suppresses virus-induced apoptosis.. J Virol.

[10] Gerrard SR, Bird BH, Albarino CG, Nichol ST (2007). The NSm proteins of Rift Valley fever virus are dispensable for maturation, replication and infection.. Virology.

[11] Gerrard SR, Nichol ST (2007). Synthesis, proteolytic processing and complex formation of N-terminally nested precursor proteins of the Rift Valley fever virus glycoproteins.. Virology.

[12] Ikegami T, Peters CJ, Makino S (2005). Rift valley fever virus nonstructural protein NSs promotes viral RNA replication and transcription in a minigenome system.. Journal of Virology.

[13] Billecocq A, Spiegel M, Vialat P, Kohl A, Weber F (2004). NSs protein of Rift Valley fever virus blocks interferon production by inhibiting host gene transcription.. Journal of Virology.

[14] Habjan M, Pichlmair A, Elliott RM, Overby AK, Glatter T (2009). NSs protein of rift valley fever virus induces the specific degradation of the double-stranded RNA-dependent protein kinase.. Journal of Virology.

[15] Ikegami T, Narayanan K, Won S, Kamitani W, Peters CJ (2009). Rift Valley fever virus NSs protein promotes post-transcriptional downregulation of protein kinase PKR and inhibits eIF2alpha phosphorylation.. PLoS Pathogens.

[16] Bouloy M, Janzen C, Vialat P, Khun H, Pavlovic J (2001). Genetic evidence for an interferon-antagonistic function of rift valley fever virus nonstructural protein NSs.. Journal of Virology.

[17] Won S, Ikegami T, Peters CJ, Makino S (2007). NSm Protein of Rift Valley Fever Virus Suppresses Virus-Induced Apoptosis.. Journal of Virology.

[18] Popova TG, Turell MJ, Espina V, Kehn-Hall K, Kidd J (2010). Reverse-phase phosphoproteome analysis of signaling pathways induced by Rift valley fever virus in human small airway epithelial cells.. PloS One.

[19] Moll UM, Wolff S, Speidel D, Deppert W (2005). Transcription-independent pro-apoptotic functions of p53.. Current Opinion in Cell Biology.

[20] Shieh SY, Ikeda M, Taya Y, Prives C (1997). DNA damage-induced phosphorylation of p53 alleviates inhibition by MDM2.. Cell.

[21] Wang P, Reed M, Wang Y, Mayr G, Stenger JE (1994). p53 domains: structure, oligomerization, and transformation.. Mol Cell Biol.

[22] Steegenga WT, van der Eb AJ, Jochemsen AG (1996). How phosphorylation regulates the activity of p53.. Journal of Molecular Biology.

[23] Shieh SY, Taya Y, Prives C (1999). DNA damage-inducible phosphorylation of p53 at N-terminal sites including a novel site, Ser20, requires tetramerization.. The EMBO Journal.

[24] Oda K, Arakawa H, Tanaka T, Matsuda K, Tanikawa C (2000). p53AIP1, a Potential Mediator of p53-Dependent Apoptosis, and Its Regulation by Ser-46-Phosphorylated p53.. Cell.

[25] Meek DW (1994). Post-translational modification of p53.. Seminars in Cancer Biology.

[26] Kohn KW (1999). Molecular interaction map of the mammalian cell cycle control and DNA repair systems.. Molecular Biology of the Cell.

[27] Thakur VS, Ruhul Amin ARM, Paul RK, Gupta K, Hastak K (2010). p53-Dependent p21-mediated growth arrest pre-empts and protects HCT116 cells from PUMA-mediated apoptosis induced by EGCG.. Cancer Letters.

[28] Haupt Y, Rowan S, Shaulian E, Vousden KH, Oren M (1995). Induction of apoptosis in HeLa cells by trans-activation-deficient p53.. Genes & Development.

[29] Kelekar A, Thompson CB (1998). Bcl-2-family proteins: the role of the BH3 domain in apoptosis.. Trends in Cell Biology.

[30] Myskiw C, Arsenio J, Hammett C, van Bruggen R, Deschambault Y (2011). Comparative analysis of poxvirus orthologues of the Vaccinia Virus E3 protein: Modulation of PKR activity, cytokine responses and virus pathogenicity..

[31] Sir D, Tian Y, Chen W-l, Ann DK, Yen T-SB (2010). The early autophagic pathway is activated by hepatitis B virus and required for viral DNA replication.. Proc Natl Acad Sci U S A.

[32] Turpin E, Luke K, Jones J, Tumpey T, Konan K (2005). Influenza virus infection increases p53 activity: role of p53 in cell death and viral replication.. Journal of Virology.

[33] Vialat P, Muller R, Vu TH, Prehaud C, Bouloy M (1997). Mapping of the mutations present in the genome of the Rift Valley fever virus attenuated MP12 strain and their putative role in attenuation.. Virus Res.

[34] Ikegami T, Won S, Peters CJ, Makino S (2006). Rescue of infectious rift valley fever virus entirely from cDNA, analysis of virus lacking the NSs gene, and expression of a foreign gene.. J Virol.

[35] Ikegami T, Narayanan K, Won S, Kamitani W, Peters CJ (2009). Rift Valley fever virus NSs protein promotes post-transcriptional downregulation of protein kinase PKR and inhibits eIF2alpha phosphorylation.. PLoS Pathog.

[36] Milczarek GJ, Martinez J, Bowden GT (1997). p53 Phosphorylation: biochemical and functional consequences.. Life Sciences.

[37] Moll UM, Petrenko O (2003). The MDM2-p53 Interaction.. Molecular Cancer Research.

[38] Zhang Y, Xiong Y (2001). A p53 amino-terminal nuclear export signal inhibited by DNA damage-induced phosphorylation.. Science (New York, NY).

[39] Vialat P, Billecocq A, Kohl A, Bouloy M (2000). The S Segment of Rift Valley Fever Phlebovirus (Bunyaviridae) Carries Determinants for Attenuation and Virulence in Mice.. Journal of Virology.

[40] Ikegami T, Narayanan K, Won S, Kamitani W, Peters CJ (2009). Dual Functions of Rift Valley Fever Virus NSs Protein: Inhibition of Host mRNA Transcription and Post-transcriptional Downregulation of Protein Kinase PKR.. Ann N Y Acad Sci.

[41] Mansuroglu Z, Josse T, Gilleron J, Billecocq A, Leger P (2010). Nonstructural NSs protein of rift valley fever virus interacts with pericentromeric DNA sequences of the host cell, inducing chromosome cohesion and segregation defects.. J Virol.

[42] Kalveram B, Lihoradova O, Ikegami T (2011). NSs protein of rift valley fever virus promotes posttranslational downregulation of the TFIIH subunit p62.. Journal of Virology.

[43] Baer A, Austin D, Narayanan A, Popova T, Kainulainen M (2012). Induction of DNA Damage Signaling upon Rift Valley Fever Virus Infection Results in Cell Cycle Arrest and Increased Viral Replication.. J Biol Chem.

[44] Kurihara A, Nagoshi H, Yabuki M, Okuyama R, Obinata M (2007). Ser46 phosphorylation of p53 is not always sufficient to induce apoptosis: multiple mechanisms of regulation of p53-dependent apoptosis.. Genes to Cells: Devoted to Molecular & Cellular Mechanisms.

[45] Popova TG, Turell MJ, Espina V, Kehn-Hall K, Kidd J (2010). Reverse-phase phosphoproteome analysis of signaling pathways induced by Rift valley fever virus in human small airway epithelial cells.. PLoS ONE.

[46] Le May N, Dubaele S, Proietti De Santis L, Billecocq A, Bouloy M (2004). TFIIH transcription factor, a target for the Rift Valley hemorrhagic fever virus.. Cell.

[47] Di Lello P, Miller Jenkins LM, Mas C, Langlois C, Malitskaya E (2008). p53 and TFIIEα share a common binding site on the Tfb1/p62 subunit of TFIIH.. Proceedings of the National Academy of Sciences.

[48] Chen X, Farmer G, Zhu H, Prywes R, Prives C (1993). Cooperative DNA binding of p53 with TFIID (TBP): a possible mechanism for transcriptional activation.. Genes & Development.

[49] Lazo PA, Santos CR (2011). Interference with p53 functions in human viral infections, a target for novel antiviral strategies?.

[50] Scheffner M, Werness BA, Huibregtse JM, Levine AJ, Howley PM (1990). The E6 oncoprotein encoded by human papillomavirus types 16 and 18 promotes the degradation of p53.. Cell.

[51] Werness BA, Levine AJ, Howley PM (1990). Association of human papillomavirus types 16 and 18 E6 proteins with p53.. Science.

[52] Pise-Masison CA, Brady JN (2005). Setting the stage for transformation: HTLV-1 Tax inhibition of p53 function.. Front Biosci.

[53] Tabakin-Fix Y, Azran I, Schavinky-Khrapunsky Y, Levy O, Aboud M (2006). Functional inactivation of p53 by human T-cell leukemia virus type 1 Tax protein: mechanisms and clinical implications.. Carcinogenesis.

[54] Yang MR, Lee SR, Oh W, Lee EW, Yeh JY (2008). West Nile virus capsid protein induces p53-mediated apoptosis via the sequestration of HDM2 to the nucleolus.. Cell Microbiol.

[55] Devireddy LR, Jones CJ (1999). Activation of caspases and p53 by bovine herpesvirus 1 infection results in programmed cell death and efficient virus release.. J Virol.

[56] Verbruggen P, Ruf M, Blakqori G, Overby AK, Heidemann M (2011). Interferon Antagonist NSs of La Crosse Virus Triggers a DNA Damage Response-like Degradation of Transcribing RNA Polymerase II.. J Biol Chem.

[57] Blakqori G, Delhaye S, Habjan M, Blair CD, Sanchez-Vargas I (2007). La Crosse bunyavirus nonstructural protein NSs serves to suppress the type I interferon system of mammalian hosts.. J Virol.

[58] Elliott RM, Weber F (2009). Bunyaviruses and the type I interferon system.. Viruses.

