# Supplementary figures and images for: p53 Activation following Rift Valley Fever Virus Infection Contributes to Cell Death and Viral Production
Source: PLoS One. 2012 May 4;7(5):e36327. doi: 10.1371/journal.pone.0036327 (PMC3344861; doi:10.1371/journal.pone.0036327)

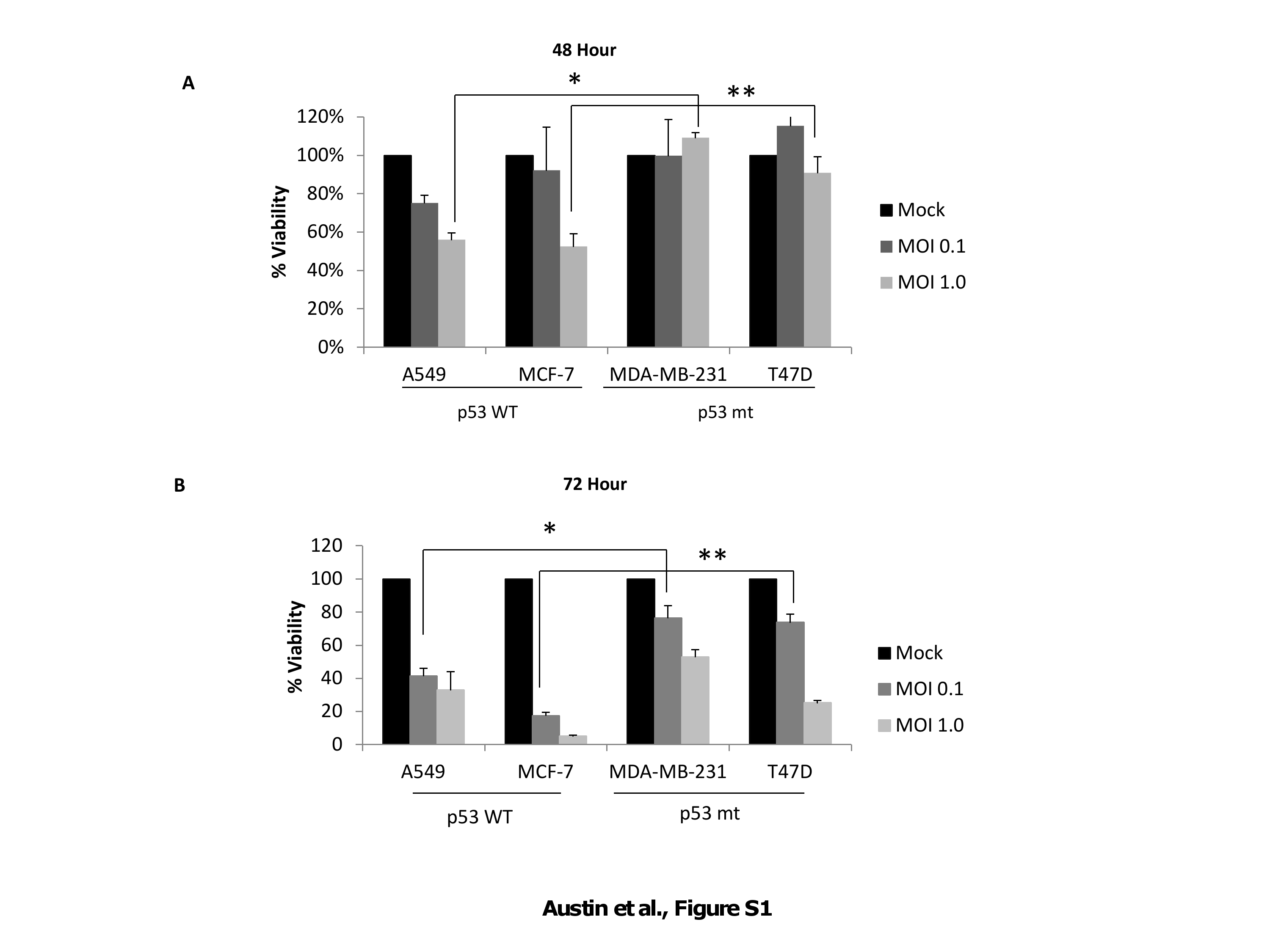

Supplement: Figure S1 — p53 mutant cells are more resistant to RVFV induced cell death. p53 WT (A549 and MCF-7) and p53 mt (MDA-MB-231 and T47D) cells were plated at 25,000 cells per well in a 96 well plate. Cells were mock infected or infected with MP-12 (MOI 0.1 and 1.0). Cell viability was determined at A) 48 or B) 72 hours post-infection by CellTiter Glo Assay (Promega). Viability of the infected cells was calculated relative to the mock infected cells (100%) (average of triplicates shown). (*) Indicates statistically significant difference (unpaired t-test of triplicates) p<0.01. Error bars indicate standard deviation. (TIF) [file pone.0036327.s001.tif]
